# Dissecting microRNA-mediated regulation of stemness, reprogramming, and pluripotency

**DOI:** 10.1186/s13619-016-0028-0

**Published:** 2016-03-22

**Authors:** Young Jin Lee, Suresh Ramakrishna, Himanshu Chauhan, Won Sun Park, Seok-Ho Hong, Kye-Seong Kim

**Affiliations:** 1 iDream Research Center, MizMedi Women’s Hospital, Seoul, 07639 South Korea; 2 Department of Biomedical Science, Graduate School of Biomedical Science and Engineering, Hanyang University, 222 Wangsimni-ro, Seongdong-gu, Seoul, 04763 South Korea; 3 College of Medicine, Hanyang University, Seoul, South Korea; 4 Uppsala University, 75236 Uppsala, Sweden; 5 Department of Physiology, School of Medicine, Kangwon National University, Chuncheon, 24341 South Korea; 6 Department of Internal Medicine, School of Medicine, Kangwon National University, 1 Kangwondaehak-gil, Chuncheon-si, Gangwon-do 24341 South Korea; 7 Stem Cell Institute, Kangwon National University, Chuncheon, 24341 South Korea

**Keywords:** miRNAs, Embryonic stem cells, Pluripotency, Reprogramming, Self-renewal

## Abstract

Increasing evidence indicates that microRNAs (miRNAs), endogenous short non-coding RNAs 19–24 nucleotides in length, play key regulatory roles in various biological events at the post-transcriptional level. Embryonic stem cells (ESCs) represent a valuable tool for disease modeling, drug discovery, developmental studies, and potential cell-based therapies in regenerative medicine due to their unlimited self-renewal and pluripotency. Therefore, remarkable progress has been made in recent decades toward understanding the expression and functions of specific miRNAs in the establishment and maintenance of pluripotency. Here, we summarize the recent knowledge regarding the regulatory roles of miRNAs in self-renewal of pluripotent ESCs and during cellular reprogramming, as well as the potential role of miRNAs in two distinct pluripotent states (naïve and primed).

## Background

MicroRNAs (miRNAs) are endogenous short non-coding RNAs 19–24 nucleotides in length that regulate gene expression at the post-transcriptional level [1]. The first miRNA was identified in *C. elegans* by Lee and colleagues [2], who demonstrated that the lin-4 miRNA downregulated the expression of the LIN-14 protein via an antisense RNA-RNA interaction [2]. Since the term miRNA was coined in 2001 [3], numerous miRNAs have been identified in various organisms from plants to mammals. Further, miRNAs are evolutionarily conserved and are thus recognized as one of the essential regulators in the control of many different processes including development, homeostasis, and metabolism [4]. In addition, aberrant miRNA expression is involved in several diseases including cancer and chronic obstructive pulmonary disease [5, 6]. Because each miRNA targets a large number of mRNAs and multiple miRNAs can bind to one specific mRNA, the potential impact of miRNAs on the expression of a large number of proteins and on transcriptome regulation is increasingly being investigated to determine the crucial role of miRNAs in various biological events.

Recent findings have revealed that molecular mechanisms underlying the maintenance of embryonic stem cell (ESC) pluripotency and cellular reprogramming have been linked to miRNAs [1, 7]. ESCs are pluripotent cell lines derived from the inner cell mass of blastocysts [8, 9] and are characterized by two major properties that define them: an unlimited self-renewal capacity in vitro and pluripotency. Also, ESCs are able to form all three germ layers and give rise to all cell types in the tissues of the body [8, 9]. Due to these important properties, ESCs represent a valuable tool for disease modeling, drug discovery, developmental studies, and potential cell-based therapies in regenerative medicine [10, 11]. A complex set of intrinsic and extrinsic factors regulate the balance between self-renewal and lineage commitment in ESCs [12]. However, several aspects regarding the proliferation and differentiation of ESCs at the post-transcriptional level remain unknown. Numerous studies have described the expression of unique clusters of miRNAs in ESCs including miRNAs in the human and mouse miR-302 clusters, the mouse miR-290 cluster, and the human miR-371 cluster [7, 13]. It has been definitively predicted that miRNAs may be a valuable means to regulate the proliferation and the differentiation of ESCs. Here, we have reviewed the recent discovery of miRNAs in ESCs and ESC-like stem cells and their role in the regulation of self-renewal and during cellular reprogramming.

## Biogenesis and biological action of microRNAs (miRNAs)

MiRNAss regulate gene expression at the post-transcriptional level by binding to the 3′-untranslated regions (3′ UTRs) or the open reading frames of target genes, resulting in the degradation of target mRNA or the inhibition of mRNA translation [1, 4]. MiRNAs represent ~4 % of the genes in the human genome and regulate the expression of more than one third of the protein-coding genes at the post-transcriptional level [14]. Gene expression is controlled by mRNA sequestration, translation repression, or miRNA-mediated mRNA decay [15]. Approximately half of miRNA genes are located in intergenic regions and can be controlled from their own promoters or as polycistronic clusters from a shared promoter, whereas the remaining miRNAs are embedded within protein-coding genes and are co-transcribed with their host genes or from miRNA-specific promoters [1, 4, 16]. Mature miRNAs are generated by multiple sequential endonucleolytic cleavage steps. The microprocessing complex consists of the RNAse III-like enzyme Drosha and its cofactor DiGeorge syndrome critical region gene 8 (DGCR8) [17–22]. Pre-miRNAs are further cleaved by Dicer, an RNAse III enzyme, which gives rise to a double-stranded RNA 22–24 nucleotide comprised of the mature miRNA (guide strand) and the miRNA passenger strand [23, 24]. Subsequently, the double-stranded mature RNA with a less thermodynamically stable 5′-end (the guide strand) is recruited by Argonaute proteins (AGOs) and is loaded into the RNA-induced silencing complex (RISC) to act as an miRNA [25, 26]. The RISC acts as an effector that facilitates miRNA-dependent silencing via binding of miRNAs to the 3′ UTR of the target mRNA transcript based on complementarity between the miRNA and the miRNA target. Nucleotides 2–8 (from the 5′ end) of the mature miRNA (“seed region”) are crucial for target identification, as perfect complementarity guides the miRNA-induced degradation of the target mRNA through AGO2 endonuclease activity [27, 28]. Partial pairing results in repression of the target mRNA translation at the initiation step, or in sequestration of the target mRNAs into cytoplasmic processing bodies, which happens by engaging poly(A) nucleases to degrade mRNA through deadenylation pathways [29].

## Embryonic stem cell (ESC)-specific microRNAs (miRNAs)

The molecular basis of miRNAs for mouse ESCs (mESCs) was initially demonstrated in mESCs lacking Dicer and Dgcr8 [30–32]. Although Dicer- or Dgcr8-deficient mESCs are viable, Dicer or Dgcr8 loss compromises the biogenesis of miRNA and causes severe defects in the proliferation and differentiation of mESCs. Furthermore, Dicer- and Dgcr8-deficient mESCs fail to generate detectable teratomas and chimeric mice when subcutaneously injected into nude mice and into blastocysts [30, 33]. These findings demonstrated the importance of miRNA synthesis in mESC pluripotency and during early embryonic development [34].

In parallel with studies on the role of essential factors for miRNA biogenesis in ESCs, the identification of ESC-specific miRNAs has been explored in mESCs and human ESCs (hESCs) to understand the post-transcriptional regulation of genes related to the self-renewal and pluripotency of ESCs [34]. Several techniques including cloning, qPCR, microarray, and deep sequencing have been employed to examine the expression of miRNAs in undifferentiated ESCs and their differentiated counterparts. Interestingly, only a few ESC-enriched miRNAs are transcribed and are unique to ESCs, whereas other miRNAs are widely expressed but decrease dramatically during differentiation. Thus, ESCs are comprised of a unique set of ESC-specific miRNAs [7, 35–37].

A large portion of these ESC-specific miRNAs constitute two clusters: the miR-290 cluster in mice and their human homologs in the miR-371-373 family and the miR-302-367 cluster in both mice and humans [7]. Later, Suh et al. identified several novel miRNAs from an undifferentiated human ESC cDNA library belonging to the miR-302 and miR-371 clusters [13]. In comparison with Houbaviy’s data set, there are three common miRNAs (miR-296, miR-301, and miR-302) between the mESCs and hESCs data sets. Taken together, these findings strongly suggest that combinatorial regulation of ESC-specific miRNAs and their target networks plays a critical role in the maintenance of ESC pluripotency and in the regulation of early embryonic development.

## Role of embryonic stem cell (ESC)-specific microRNAs (miRNAs)

Substantial evidence has shown that the miRNAs clusters miR-302, miR-209, and miR-371 represent key regulators of pluripotent stem cells [34, 38]. All of these miRNAs clusters play critical roles in cellular processes, such as maintaining pluripotency, proliferation, and differentiation, which are the characteristics of stemness. Recent findings suggest that ESC-specific transcription factors regulate ESC-specific miRNAs and, in turn, these miRNAs control transcription factors, suggesting that ESC-specific miRNAs are an essential and integral part of the ESC dynamics [34, 39]. Furthermore, several reports of functional investigations related to expression and inhibition of miRNAs in ESCs suggest that miRNAs are one of the key factors that control stemness in ESCs [40, 41]. This applies to ESCs in which miRNAs might be used as a tool to control proliferation and differentiation.

### Function of miR-302 cluster members

The high degree of homology, regulation by ESC-specific transcription factors, conservation of genomic loci, and cell type-specific expression could be the basis for the functional conservation of the miR-302 cluster in ESCs. The ESC cell cycle is significantly shorter than that of somatic cells, due largely to an abbreviated G1 phase [42]. In terms of cell cycle regulation, the ectopic expression of individual miR-302 members in both primary and transformed cell lines leads to a decrease in the proportion of cells in the G1 phase and an increase in the proportion of cells in the S phase by the inhibition of Cyclin D1 translation, which is a G1 phase regulator. This evidence suggests that Cyclin D1 might be targeted by multiple members of the miR-302 cluster, and that one of the primary functions of miR-302 in ESCs is cell cycle regulation [43].

For the maintenance and differentiation of ESCs, miR-302b indirectly regulates Oct4 and directly targets Cyclin D2, which is an important developmental regulator during gastrulation, suggesting that miR-302b participates in maintaining the pluripotency of embryonic carcinoma cells (ECCs) [44]. Prior reports have indicated that the TGF-β/Nodal signaling pathway is critical to the maintenance of pluripotency in hESCs [45, 46]. Lefty1 and 2, which belong to the TGF-β/Activin/Nodal family and act as primary antagonists of Nodal signaling, were negatively regulated at both the transcriptional and translational levels by miR-302-367 [47]. Thus, the ectopic expression of miR-302-367 might act as upstream regulators of the TGF-β/Nodal signaling pathway via Smad-2/3 signaling, leading to a delay in early hESC differentiation and facilitating the maintenance of pluripotency in hESCs [48]*.* Lipchina et al. identified several putative targets of miR-302-367 in hESCs by genome-wide screening processes, which suggested that this miRNA cluster is a positive regulator of pluripotency [49]. Other studies showed that miR-302-367 is capable of regulating bone morphogenetic protein (BMP) signaling in hESCs during neural differentiation [50], and that the inhibition of this cluster leads to reduced efficiency of BMP-dependent trophectoderm induction, which suggests that miR-302-367 acts as a positive regulator of BMP signaling [49, 51]. Recently, miR-302 cluster-silenced mouse embryos exhibited defects in neural differentiation by inhibition of neural progenitor expansion and precocious differentiation [52]. In regards to reproduction, Scheel et al. identified the miR-302 cluster as a potential suppressor of p63 accumulation in various cell types by running a functional screen utilizing a large miRNA expression library. The mRNA and protein expression levels of p63 were reduced by miR-302 via two target sites within the 3′ UTR. In addition, miR-302 displayed regulatory mechanisms for p63 in germ cells by suppressing p63 in testicular cancer cells and eliminating p63 mRNA in mature oocytes [53].

### Function of miR-290 cluster

Members of the miR-290 cluster are the most highly expressed miRNAs in mESCs comprising more than 60–70 % of the total miRNAs expressed and are studied extensively in various backgrounds [54] including DNA methylation, maintenance of pluripotency, germ cell development, and generation of induced pluripotent stem cells (iPSCs) [55, 56].

It was demonstrated that the miR-290 cluster was involved in silencing the expression of Rbl2, which acts as a transcriptional repressor of the DNA methyltransferase (Dnmt) 3a and Dnmt3b enzymes. Further, Dnmt3a and Dnmt3b enzymes, along with miRNAs, are downregulated in Dicer1−/− cells [57]. Another research group showed a mechanistic connection between members of the miR-290 group and de novo DNA methylation in ESCs, providing a clue that these miRNAs are involved in the epigenetic control of gene expression. This de novo DNA methylation is defective in Dicer1-deficient ESCs, which is consistent with the indirect control of Dnmt expression by the miR-290 cluster [56]. Meanwhile, the miR-290 cluster inhibits the arrest of ESCs in the G1 phase by suppressing several key regulators of the G1-S transition, thus accelerating cell proliferation by promoting the G1 to S phase transition [58]. Additional research on cell cycle dynamics has shown that miR-290 and let-7 miRNAs have opposing effects on ESC characterization of self-renewal and pluripotency [59–61]. It is assumed that these two miRNA clusters exhibit a feedback regulatory cycle, which allows a quick exchange mechanism between self-renewal and differentiation of ESCs. The necessity for miRNA biogenesis during primordial germ development and early spermatogenesis was established by the demonstration of increased expression of miR-290 cluster members in male germline cells [62]. It was reported that Dgcr8−/− ESCs failed to differentiate into germ layers, and the expression levels of pluripotent marker genes including Oct4, Sox2, and Nanog were increased in Dgcr8-deficient ESCs, indicating that these pluripotent markers are indeed in control of miRNAs [63]. In addition, the miR-290 cluster showed some evidence of Wnt signaling regulation by repressing the Wnt pathway inhibitor Dkk-1 and by preventing ES cell differentiation to mesoderm and germ cells in vitro [64]. In a loss-of-functional study, the partial lethality phenotype was observed in embryos with a homozygous loss of the miR-290–295 locus that also resulted in infertility among female survivors, suggesting that some of the members could be responsible for the maintenance of pluripotency [65]. Recently, Kanellopoulou et al. suggested that one of the miR-290 families of miRNAs (miR-291) regulates ESC homeostasis by silencing polycomb-mediated gene expression via targeting methlytransferase Ash1l in ESCs [66].

### Function of miR-371 cluster

MiR-371 is an orthologous human cluster of the mouse miR-290 family (miR-290-295) that is preferentially expressed in hESCs and that decreases rapidly in expression after differentiation [13, 37]. Although the contributions of this miRNA cluster to gene expression programs in pluripotent stem cells have not been fully elucidated, Cao et al. recently reported that miR-290/371 promotes pluripotency by regulating glycolytic metabolism via the Mbd2-Myc signaling pathway in mouse and human ESCs [67]. Furthermore, this miRNA cluster is one of the ESC-specific miRNA clusters, but a few reports have also described the involvement of miR-371 in the regulation of embryonic cell carcinoma (ECC) and tumorigenesis [68]. The expression of the miR-372/373 cluster was observed in subsets of ECC lines including Tera1, 2102Ep, and 833KE, but not in NT2 and NCCIT cell lines [69], which were correlated with the expression of the miR-372/373 cluster and p53 status in these ECC lines, based on low-level wt-p53 expression in NT2 cells and single mutated alleles in NCCIT lines. In light of these results, it was suggested that miRNAs in the miR-372/373 cluster contribute to tumorigenesis in cells that contain wt-p53 [69]. It has been demonstrated that cisplatin represses the oncogenic properties of the miR-372/373 group [70] and that miR-373 and miR-520 trigger migration and invasion of cancer cells in vitro and in vivo [71]. In regards to gain-of-functional studies, the over-expression of miR-373 instantly induced the expression of the E-cadherin and cold-shock domain-containing protein C2 (CSDC2) genes, and the induction was specifically dependent on the presence of both miR-373 and the proposed target sites of miR-373 within the promoter region of the E-cadherin and CSDC2 genes [72].

## Potential roles of microRNAs (miRNAs) in naïve or primed pluripotency

In mouse, ESCs are derived from the inner cell mass (ICM) of blastocysts (naïve mESCs) and have different characteristics in gene expression profiles, signaling pathways for maintaining pluripotency, and in the development potential compared to primed mESCs derived from the epiblast of post-implantation embryos (mEpiSCs) [73–77]. In human, ESCs are established from the ICM of preimplantation blastocysts, but these hESCs more closely resemble primed mESCs (mEpiSCs) than naïve mESCs when assessed based on the characteristics mentioned above [78–80]. In addition, mouse and human induced pluripotent stem cells (iPSCs) derived by introducing transcription factors into somatic cells have been shown to have the same characteristics as primed ESCs [81].

Regarding the link between pluripotent states (naïve or primed) and microRNAs, the miR-290/295 cluster is a major miRNA in mouse ICM and ESCs [82] and upon implantation, expression of miR-302/367, miR-25/106b, miR-17/92a, and miR-106a/363 clusters is restricted to epiblasts [83]. In addition, the miR-20, miR-92, and miR-302 miRNA seed families regulate primed pluripotent stem cell survival via targeting the pro-apoptotic protein BIM [83]. Expression of the miR-302/367 cluster is enough to drive the reprogramming of murine and human somatic cells to a naïve or primed pluripotency in the absence of exogenous key transcription factors [84]. Jouneau et al. reported that mESCs and mEpiSCs exhibit a different miRNA expression profile with a different set of pluripotent-associated miRNAs by deep sequencing of small RNA libraries from three primed mEpiSCs and two naïve mESCs. Also, this report indicate that 302 of 987 mature miRNAs are differentially expressed in each pluripotent stem cell state, and among them, miR-302d, miR-34c, miR-367, and let-7e are more highly expressed in EpiSCs, whereas miR-294 and miR-142-3p are preferentially expressed in mESCs. The five members of the miR-200 family (miR-200a, miR-200b, miR-200c, miR-141, and miR-429) are expressed in mESCs and downregulated during epithelial-mesenchymal transition or EMT. Further, expression of miR-200c/141 suspends the differentiation of mESCs in an EpiESC-like state [85].

In human pluripotent stem cells, miR-302b, miR-372, miR-518b, miR-520b, and miR-520c are expressed in human primed pluripotent ESCs, and the primate-specific chromosome 19 miR cluster (miR-518b, miR-520b, and miR-520c) is expressed in primed state pluripotent cells but declines upon differentiation [37, 86]. In addition, miR-302b is a positive control for naïve and primed human pluripotent stem cells, whereas miR-518, miR-520b, and miR-520c are upregulated during a shift to an earlier pluripotency state [87]. Recently, Zhang et al. reported that porcine-induced pluripotent stem cells (piPSCs) were established by manipulating the culture conditions (treatment of LIF, FGF2, and BMP4 with 2i (CHIR99021 and SB431542)). Zhang et al. also reported that these induced cells exhibited mixed miRNA profiles, including upregulation of the miR-302b/367 and miR-106a/363 clusters, and downregulation of let-7 family members and the miR-17/92 cluster [88].

Although there are several reports that include the expression profiles of miRNAs in naïve and primed pluripotent stem cells, studies examining the functional roles of these miRNAs that make distinctions between naïve and primed pluripotent stem cells have not been performed satisfactorily. Therefore, the physiological roles of miRNAs in regulating pluripotent stages need to be determined.

## Importance of microRNAs (miRNAs) related to induced pluripotency

Somatic cells could be transformed to iPSCs through epigenetic reprogramming by ectopic expression of key transcription factors [81]. However, genomic modifications due to random insertions of exogenous DNA into the host genome remain a concern. To address this concern, several research groups have tried to generate iPSCs from somatic cells using PiggyBac transposons, episomal systems, or proteins or mRNAs [89–93], but these methods were technically challenging due to the low efficiency of the processes for inducing pluripotency.

Recently, miRNAs have been used to reprogram primary somatic cells toward pluripotent cells [41, 94–97]. Further, ESC-specific miRNAs that are preferentially expressed in ESCs and are known to be involved in the control of pluripotent-related factors were identified. The ESC-specific miR-302 family, which is composed of five members, miR-302a/b/c/d and miR-367, is highly expressed in undifferentiated mESCs, hESCs, and iPSCs, but suppressed in differentiated cells [98]. MiR-302 regulates the expression of the pluripotency markers Oct4, Sox2, Nanog, and SSEA-3/4 [99] and stimulates somatic cell reprogramming, which results in the generation of iPSCs through DNA demethylation and by downregulation of Dnmt1 [98] and methyl-DNA binding domain protein 2 (MBD2) [100]. In addition, miR-302 facilitates mesenchymal to epithelial transition or MET via targeting of the transforming growth factor β receptor II (TGFBR2) and the Ras homolog gene family member C genes [95]. Recently, Zhang et al. reported that knockout of the miR-302/367 cluster completely blocks human iPSC generation from human foreskin fibroblasts [101]. Therefore, the miR-302 family plays important roles in the reprogramming of somatic cells for iPSC generation. Further, several miRNAs enhance genomic reprogramming to induce iPSCs by combined expression with key transcription factors (Oct4, Sox2, Klf4, and Myc; OSKM) [102]. The miR-290 family (miR-371 in humans) plays essential roles during the reprogramming progress. Mir-291-3p, miR-294, and miR-295 (miR-290 family) have been shown to increase the efficiency of reprogramming by OSK and ectopic expression of this cluster improves OSKM- or OSK-reprogramming by inhibition of the TGF-β receptor signaling [94–96, 102].

Reprogramming technologies are thought to be one of the solutions for treating many age-associated diseases; thus, several research groups are attempting to cure diseases using iPSCs [103]. For example, despite the high resistance to reprogramming apparent in old cells or tissues, Sharma et al. reported that over-expression of Sirtuin 6 (SIRT6), which is one of the miR-766 targets in aged human dermal fibroblasts, increases iPSC generation efficiency through the control of miR-766 transcription via feedback regulation [104]. Wang et al. found that Oct4 or Sox2 bind at the promoter region of members of the miR-200 family of miRNAs, and that these miRNAs help Oct4/Sox2 induce somatic cell reprogramming in the early stage by direct inhibition of zinc finger E-box binding homeobox 2 (ZEB2) [105]. Members of the miR-200 family are also involved in the regulation of EMT induction as well as MET [106–108]. In addition, epigenetic regulation of the miR-200 family results in the conversion of a non-stem to a stem-like cell by the loss of their expression [109]. The miR-181 family consists of four miRNAs (miR-181a, miR-181b, miR-181c, and miR-181d) and these miRNAs act as tumor suppressors in human malignant glioma [110] or as activators of carcinogenesis in hepatic cancer, blood cancers, and breast cancer [111–116]. In terms of cell differentiation, members of the miRNA-180 family of miRNAs inhibit the differentiation of hematopoietic cells to mature cells via p27 targeting [117, 118]. Interestingly, the miR-180 family of miRNAs is upregulated following the introduction of key pluripotency factors (Oct4, Sox2, and Klf4) and target several signaling molecules (Lin7c, Tox, Bptf, Marcks, etc.), which results in the enhanced generation of iPSCs [119]. Also, several studies have shown that the expression profiles of miRNAs were different in hESCs, iPSCs, differentiated cells, and cancer cells [120–122], which provides us with the fundamental knowledge for developing another way to induce iPSCs using preferentially expressed miRNAs without genomic modification.

It is well known that p53 is a reprogramming barrier for iPSC generation [123–126] and that several p53-related miRNAs were identified as modulators or mediators of iPSC generation. The p53 signaling pathways are regulated by mir-21 and -29b, and Sox2 regulates miR-29b expression, which suppresses DNA methylation-related reprogramming events (e.g., MET and Dlk1-Dio3 region transcription), resulting in enhanced iPSC generation [127, 128]. MiR-34 is one of the p53 targets that contributes to p53 repression of iPSC generation and that exhibits p53-dependent stimulation during reprogramming [129]. The depletion of miR-34 results in successful iPSC generation without compromising cell dynamics; therefore, miR-34 plays a crucial role in inhibiting somatic reprogramming. As targets of p53, miR-92 and miR-141 have distinguishable expression profiles in hESCs and iPSCs, and they provide two distinct pluripotency categories irrespective of the cell origin [121]. Finally, miR-138 directly targets the 3′ UTR of p53, resulting in suppression of p53 expression and its downstream genes, and significant improvement in iPSC generation [130]. Furthermore, the miR-17-92, miR-106b-25, and miR-106a-364 clusters are highly induced in the early stages of somatic cell reprogramming and directly target p21 and TGFBR2, resulting in enhanced iPSC generation by accelerating MET, cell cycle transitions, and regulation of epigenetic factors [94, 96].

Here, we summarized the interaction of miRNAs with their targets in DNA methylation, MET, and the cell cycle for somatic cell reprogramming (Table 1). Although pluripotency transcription factors are known to bind at several regulatory regions for miRNA transcription, or to inhibit their expression during somatic cell reprogramming, the precise mechanisms by target genes of these miRNAs remain unknown and require additional study for high efficiency iPSC generation and their clinical applications in the future.

**Table 1:** miRNAs involved in somatic reprogramming

miRNAs	Effect on reprogramming	Target	Reference
miR-200s	Stimulation	ZEB2	[105]
miR-181	Stimulation	Lin7c, Tox, Bptf,	[119]
		Cpsf6, Dnaj13,	
		Nol8, Cdyl, Marcks,	
		Igh2bp2, Ywhag,	
		Bclaf1, Nlr2c2	
miR-29b	Stimulation	Dnmt3a and 3b	[127, 128]
miR-17-92,	Stimulation	Tgfbr2, p21	[94, 96]
miR-106b-25,			
miR-106a-364			
miR-138	Stimulation	P53	[130]
miR-291, miR-294,	Stimulation	Brp44l, Cdkn1a, Cfl2,	[98, 100, 101, 132]
miR-295,		Ddhd1, Dpysl2, Hivep2,	
miR-302a/b/c/d,		Lefty, Mbd2, Nr2f2,	
miR-367		Pten, RhoC, Tgfbr2	
miR-766	Inhibition	SIRT6	[104]
miR-21	Inhibition	P85α, Spry1	[128]
miR-34a	Inhibition	Nanog, Sox2, N-myc	[129]

## Conclusions

**Fig. 1:**
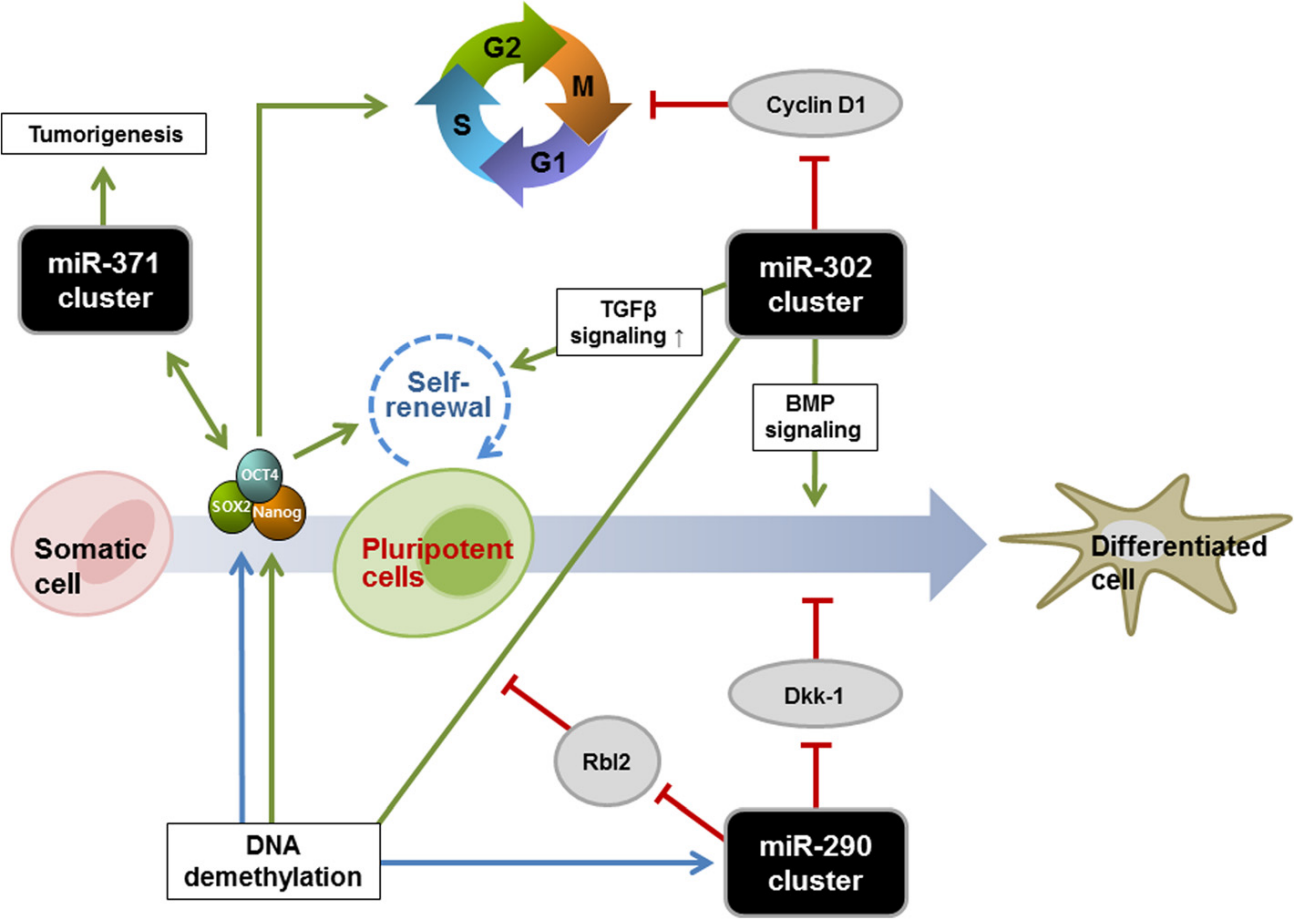
Functions of microRNAs (miRNAs) in embryonic stem cell (ESC) self-renewal, differentiation, and cellular reprogramming. ESC-specific miRNAs are involved in the maintenance and differentiation of ESCs by regulating key pluripotency factors (Oct4, Sox2, and Nanog), signaling molecules, and cell cycle distribution. Furthermore, these miRNAs play important roles in somatic cell reprogramming to gain pluripotency via epigenetic regulations

Increasing evidence suggests that miRNA regulation and function in pluripotent stem cells and somatic cell reprogramming is vital for the advancement of regenerative medicine. Over the past decade, a large number of miRNAs have emerged as pivotal components of a complex molecular network of gene expression related to the pluripotent cell state (Fig. 1).

First, can any new miRNAs be identified? Emerging advancements such as deep sequencing can be utilized to predict many new miRNAs in pluripotent stem cells. The new methods can likewise help us to directly profile the activities between miRNAs and mRNAs. Second, what is the best possible prerequisite miRNA dose for regulation of pluripotency? Discovering specific miRNA dosages might provide a possible mechanism for optimizing and standardizing final gene products and induced pluripotent products. Third, do mouse miRNAs and human miRNAs possess the same functions? Despite these open-ended questions, several studies have shown that mouse and human cell science share a great deal of similarities and that mESCs and hESCs exhibit contrasting differences in the regulation of pluripotency and further maintenance of stemness. Moreover, it is understood that hESCs are more like epiblast stem cells compared with mESCs, whereas mESCs are more “embryonic” than their human counter-partner [131]. Regardless of their indistinguishable characteristics, these reasons compel us to focus on mouse miRNAs and their human counterparts.

Altogether, there are numerous different classes of non-coding RNAs that may likewise assume a role in managing pluripotency and differentiation. One may speculate that miRNAs are involved in the fine-tuning of their targets instead of in the regulation of on/off decisions. This is because knockout of a single miRNA does not typically affect embryonic development. One of the reasons could be that a particular miRNA might be compensated for by family members due to high sequence homology. Taken together, intensive screening for targets that are regulated by miRNAs in hESCs, as well as superior insight into the regulatory and functional strategies for controlling hESC self-renewal, differentiation, and iPSC generation is needed before these pluripotent stem cells can be put into clinical practice.

## Abbreviations

3′ UTR: 3′-untranslated region
AGOs: Argonaute proteins
BMP: bone morphogenetic protein
CSDC2: cold-shock domain-containing protein C2
DGCR8: DiGeorge syndrome critical region gene 8
Dnmt: DNA methyltransferase
ECCs: embryonic carcinoma cells
ESC: embryonic stem cell
ICM: inner cell mass
iPSCs: induced pluripotent stem cells
MET: mesenchymal to epithelial transition
miRNAs: microRNAs
RISC: RNA-induced silencing complex
SIRT6: Sirtuin 6
TGFBR2: transforming growth factor β receptor II
